# A Cell-Based Assay for RNA Synthesis by the HCV Polymerase Reveals New Insights on Mechanism of Polymerase Inhibitors and Modulation by NS5A

**DOI:** 10.1371/journal.pone.0022575

**Published:** 2011-07-22

**Authors:** C. T. Ranjith-Kumar, Yahong Wen, Nielson Baxter, Kanchan Bhardwaj, C. Cheng Kao

**Affiliations:** Department of Molecular and Cellular Biochemistry, Indiana University, Bloomington, Indiana, United States of America; Saint Louis University, United States of America

## Abstract

RNA synthesis by the genotype 1b hepatitis C virus (HCV) polymerase (NS5B) transiently expressed in Human embryonic kidney 293T cells or liver hepatocytes was found to robustly stimulate RIG-I-dependent luciferase production from the interferon β promoter in the absence of exogenously provided ligand. This cell-based assay, henceforth named the 5BR assay, could be used to examine HCV polymerase activity in the absence of other HCV proteins. Mutations that decreased de novo initiated RNA synthesis in biochemical assays decreased activation of RIG-I signaling. In addition, NS5B that lacks the C-terminal transmembrane helix but remains competent for RNA synthesis could activate RIG-I signaling. The addition of cyclosporine A to the cells reduced luciferase levels without affecting agonist-induced RIG-I signaling. Furthermore, non-nucleoside inhibitor benzothiadiazines (BTDs) that bind within the template channel of the 1b NS5B were found to inhibit the readout from the 5BR assay. Mutation M414T in NS5B that rendered the HCV replicon resistant to BTD was also resistant to BTDs in the 5BR assay. Co-expression of the HCV NS5A protein along with NS5B and RIG-I was found to inhibit the readout from the 5BR assay. The inhibition by NS5A was decreased with the removal of the transmembrane helix in NS5B. Lastly, NS5B from all six major HCV genotypes showed robust activation of RIG-I in the 5BR assay. In summary, the 5BR assay could be used to validate inhibitors of the HCV polymerase as well as to elucidate requirements for HCV-dependent RNA synthesis.

## Introduction

Hepatitis C virus (HCV) infects approximately 175 million people worldwide. Approximately 50% percent of the HCV-infected individuals will develop hepatocellular carcinoma or liver cirrhosis after chronic infection [1]. Current treatment for HCV uses pegylated interferon and ribavirin, but efficacy is limited and tolerance of the treatment is a major concern, in part due to genetic predisposition [2], [3], [4].

HCV is a single-stranded RNA virus that belongs to the *Flaviviridae* family. The HCV genomic RNA is 9.6 kb in length and encodes a polypeptide, which is processed by cellular and virally-encoded proteases to generate ten structural and nonstructural proteins. The nonstructural protein 5B (NS5B) is the RNA-dependent RNA polymerase (RdRp), the catalytic subunit of the replicase complex. Based on the paradigm established with HIV/AIDS and herpesvirus, NS5B is an important target for antiviral therapy.

Several classes of NS5B inhibitors have been identified [5]. Chemically diverse non-nucleoside inhibitors have been shown to bind to one of five sites within NS5B to inhibit one or more steps in RNA synthesis [6]. Nucleotides generated from nucleoside analogs can lead to premature termination and/or errors in the viral RNA. Although several inhibitors of HCV NS5B have progressed into clinical trials, severe side effects have resulted in the discontinuation of most drug candidates [7], [8], [9]. There is a significant need to develop better drugs specific for the HCV polymerase, especially for use in combination with other therapies.

Innate immune responses provide the first line of defense against invading pathogen. Multiple, at least partially overlapping, pathways are used to detect viral infection [10]. Double-stranded RNAs and uncapped RNAs generated by viral polymerases are detected as pathogen-associated molecular patterns that are recognized by innate immunity receptors [10], [11]. Toll-like receptor 3 (TLR3) and Retinoic acid-inducible gene I (RIG-I) play important roles in detecting HCV RNAs. A spontaneous mutation in the RIG-I gene (T55I) resulted in increased HCV RNA replication in hepatocytes [12]. TLR3 is not expressed in immortalized human hepatocytes, but is expressed in primary cells from human livers and can lead to decreased HCV replication [13]. The relevance of both signaling pathways in HCV infection is further underscored by the fact that the HCV-encoded protease NS3-4A will cleave TRIF and IPS-1 (variously called IPS-1, MAVS, VISA and Cardif) adaptors for TLR3 and RIG-I, respectively, to short circuit the signaling response [14], [15], [16], [17], [18], [19].

We used signaling by the innate immune receptors RIG-I and MDA5 to develop cell-based assays for RNA synthesis by the 1 b and 2a HCV NS5B proteins in HEK 293T cells and in Huh7 cells. RNA synthesis by NS5B was found to induce RIG-I to activate luciferase reporters driven by the interferon β (IFN-β) promoter. Reporter production induced by RIG-I in this assay, to be named the 5BR assay, requires catalytically competent NS5B and is affected by NS5B association with cellular membranes. Furthermore, non-nucleoside inhibitor (NNI) from the benzothiadiazine (BTD) class of inhibitors that have previously been shown to inhibit NS5B [20], can abolish activity in the 5BR assay. The assay also reported on an interaction between the HCV NS5A protein and NS5B activity in a reaction that was helped by the C-terminal membrane-spanning helix of NS5B.

## Materials and Methods

### Constructs for expression in mammalian cells

The cDNA of RIG-I (pUNO-hRIG)) and MDA5 (pUNO-hMDA5) were from Invivogen (San Diego, CA). cDNAs to 1 b NS5B (Con1), 1a NS5B (H77) and 2a NS5B (JFH1) were amplified with specific primers and the Pfu polymerase. The cDNA was then cloned into the pUNO vector. cDNAs for NS5Bs from 3a (S52), 4a (ED43), 5a (SA13) and 6a (6a33) were synthesized by Biobasics Inc. (Markham Canada). An AgeI restriction site was added the 5′ terminal sequence of the cDNA while the codons for six histidine, a termination codon and a NheI restriction site were added to 3′ of the cDNA. The cDNA was subcloned into pUNO vector. Mutants were generated by site directed mutagenesis using the Quickchange mutagenesis kit (Agilent Technologies, Santa Clara, CA). All constructs were confirmed to have the correct sequence by DNA sequencing using the BigDye® Terminator v3.1 Cycle Sequencing Kit (Applied Biosystems, Carlsbad CA, USA). Huh7 cells were as described in Chinnaswamy et al. [21] and it was originally obtained from C.M. Rice [22]. The HEK 293T cells were from the ATCC and was cultured as described in Ranjith-Kumar et al. [23].

### Cell-based reporter assays

The RIG-I reporter assay was performed as per Ranjith-Kumar et al. [23], [24]. Plasmids expressing NS5B were co-transfected along with plasmids to express RIG-I or MDA5, as well as two luciferases: the firefly luciferase driven from an interferon-β promoter and a *Renilla* luciferase driven from a thymidine kinase (TK) promoter. For Huh7 cells the *Renilla* luciferase used a CMV promoter. Inhibitors were added 4 h after plasmid transfection. Where an exogenous RIG-I agonist was used, 3PdsR24 (a ds RNA of 24 base pairs with 5′ triphosphate) was transfected into cells at a 50 nM 24 h after the introduction of the plasmids. TLR3 assays were performed as previously described [25] with ISRE-Luc as the reporter plasmid. TLR3 expressing cells were induced with poly(I:C) at 1 µg/ml.

### In vitro RdRp assay

The HCV RdRp assay was performed as per Ranjith-Kumar et al. [26]. A standard assay consisted of 1 pmole of RNA template and 0.04 µM of recombinant HCV polymerase in a 20 µL reaction. The final buffer contained 20 mM sodium glutamate (pH 8.2), 20 mM NaCl, 4 mM MgCl_2_, 12.5 mM dithiothreitol, 0.5% (v/v) Triton X-100, 200 µM GTP, and 100 µM each of ATP and UTP, and 250 nM α-[P^32^]-CTP (Amersham, Inc.). The reactions were incubated at 25^o^ C for 60 min and stopped by phenol/chloroform extraction followed by ethanol precipitation in the presence of 5 µg of glycogen and 0.5 M ammonium acetate. The products were separated by electrophoresis on denaturing (7.5 M urea) polyacrylamide gels. The gels were wrapped in plastic, and radiolabel was quantified using a PhosphorImager (Molecular Dynamics).

#### Subcellular fractionation

The differential fractionation protocol of Ramsby et al. [27] was used to determine the approximate location(s) of the NS5B, and the C-terminally truncated 1bΔ21 version of NS5B. HEK 293T cells were transfected for 24 h to express the desired proteins. The cells were then harvested, washed with 1X PBS and permeabilized with PBS containing 0.019% digitonin, 250 mM sucrose and a protease inhibitor mix (P8340, Sigma Aldrich, St. Louis, MO). After a 15 min incubation on ice, the solution was centrifuged at 13,000×g for 15 min at 4°C. The resulting supernatant that contains cytoplasmic proteins was carefully collected and stored on ice until use. The pellet was washed with PBS, resuspended in PBS containing 0.5% Triton X-100 and protease inhibitors and stored on ice for 30 min to solubilize membrane-bound proteins. The sample was then centrifuged at 13,000×g for 15 min at 4°C to separate the membrane-associated materials and the unsolubilized materials from the cytoskeleton and nucleus. The latter was washed once with PBS and suspended in 1X Laemmli sample buffer [28]. All samples were subjected the SDS-PAGE with reducing agent. The gels used for Western blots were probed with monoclonal antibody specific to NS5B (Enzo Life Sciences; Switzerland) and NS5A (Santa Cruz Biotechnology; Santa Cruz, CA). Co-immunoprecipitation experiments to determine NS5A-5B interaction were performed according to the protocol in Qi et al. [29].

## Results

### Cells expressing HCV NS5B can induce RIG-I signaling

**Figure 1:**
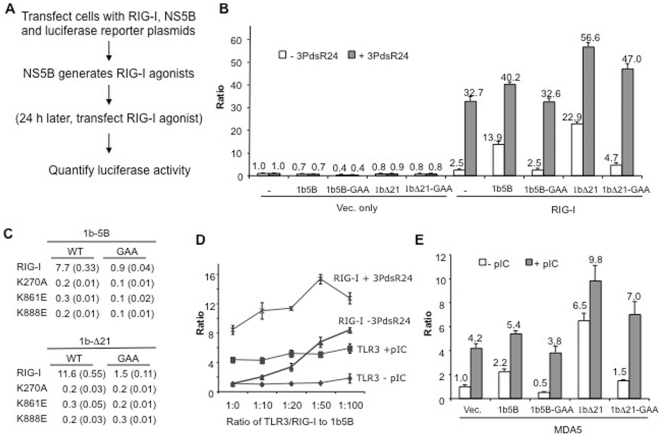
A cell-based assay for the HCV 1b polymerase. A) Schematic for the 5BR assay. The third step in the protocol, identified in parenthesis, is designed to confirm that a treatment acted through the HCV polymerase rather than the RIG-I signaling pathway. It was left out in some assays. B) Results for the 5BR assay demonstrating that expression of the 1b HCV polymerase can induce RIG-I-dependent luciferase production in HEK 293T cells. Ratio in the vertical axis denotes the units of firefly luciferase driven from the interferon β promoter relative to the *Renilla* luciferase driven from the TK promoter. The cells were transfected to express the NS5B construct denoted below the horizontal axis along with either an empty pUNO vector (Vec. only) or pUNO-RIG-I (RIG-I). The white bars show the ratios of the two luciferases in the absence of exogenously provided ligands. The grey bars show the results from cells transfected with 3PdsR24, an agonist of RIG-I. The numbers above the bars show the mean of at least three independent trials and error bars show standard deviation. The RIG-I ligand, 3PdsR24, was transfected into cells at 50 nM final concentration and serves as a check for whether RIG-I is responsive to an agonist. C) Mutations in the RIG-I protein will abolish the output in the 5BR assay. The ratios of the firefly and *Renilla* luciferases with standard deviations in parentheses are shown. D) TLR3 co-expressed in HEK 293T cells did not respond to RNA synthesis by NS5B. Effects of 1b5B on RIG-I and TLR3 signaling in the presence and absence of agonists are shown in the graph. Where used, poly(I:C) (labeled as pIC in the graph) was added to the medium of cells to a final concentration of 1 µg/ml, and the RIG-I agonist 3PdsR24 was transfected at 50 nM. E) NS5B can also induce MDA5 to activate luciferase reporter production. pUNO-MDA5 was co-transfected into the HEK 293T cells along with the plasmids expressing the RNA synthesis competent or incompetent NS5Bs. Poly(I:C) was transfected into the cells to serve as an exogenously-provided agonist to MDA5.

We seek to use innate immune signaling to develop a cell-based assay to analyze RNA synthesis by the HCV polymerase. A typical assay used HEK 293T cells transfected with four plasmids to express RIG-I, NS5B, and two luciferases that report from the IFN-β promoter and a thymidine kinase promoter (Fig. 1A). The latter serves to control for transfection efficiency and cellular toxicity. The typical assay yielded a ca. four- to nine-fold increase in the IFN-β luciferase signal when the cells were transfected with 1b5B (NS5B of genotype 1b) and a six- to twelve-fold increase was observed with the 1bΔ21, which lacks the C-terminal transmembrane helix of 21 residues (1bΔ21) (Fig. 1B). When cells expressing RIG-I and 1b5B were further transfected with the RIG-I agonist 3PdsR24, an additional increase in luciferase activity was observed (Fig. 1B), indicating that the activity induced by NS5B had not reached saturation under these conditions.

Catalytically defective mutants of 1b5B and 1bΔ21, where the divalent metal binding motifs of GDD was mutated to GAA, did not show RIG-I activation (Fig. 1B), demonstrating that RNA synthesis by NS5B was required for RIG-I activation. Furthermore, cells expressing 1b5B but not RIG-I had reporter production at background levels (Fig. 1B). Taken together, these results suggest that the RNA synthesized by NS5B was recognized by RIG-I to induce interferon-activated genes in HEK 293T cells.

Several RIG-I mutants were tested to examine whether signaling was through RIG-I. Mutant K270A, with a substitution at the ATP binding domain [30], and K861E and K888E, both in the C-terminal regulatory domain (CTD) and defective for binding RNA agonists [31], failed to be activated by 1b5B or 1bΔ21, demonstrating that signaling-competent RIG-I is required (Fig. 1C). Cells that expressed TLR3 did not alter the reporter levels, perhaps due to the RNAs generated by NS5B being inaccessible to the endosomal TLR3 (Fig. 1D). MDA5, a member of the RIG-I-like receptor family that responds to longer dsRNAs than RIG-I, was activated in the presence of 1b5B (Fig. 1E) [24], [32]. Signaling through MDA5 was less robust than that through RIG-I, but GAA mutants of 1b5B or 1bΔ21 prevented signaling. The fact that both RIG-I and MDA5 were activated suggests that at least some RNAs generated by the recombinant 1b5B fulfill the molecular patterns required for recognition by these receptors, including the lengths of the RNAs [24], [32]. Henceforth the assay where reporter activity was induced by RIG-I will be referred to as the 5BR assay. We have performed parallel assays with MDA5 for almost all of the conditions examined and found consistent results with those from the 5BR assay. For clarity, most of these results will not be shown except where we are trying to establish precedent.

### RNA synthesis by NS5B in vitro and results from the 5BR assay

**Figure 2:**
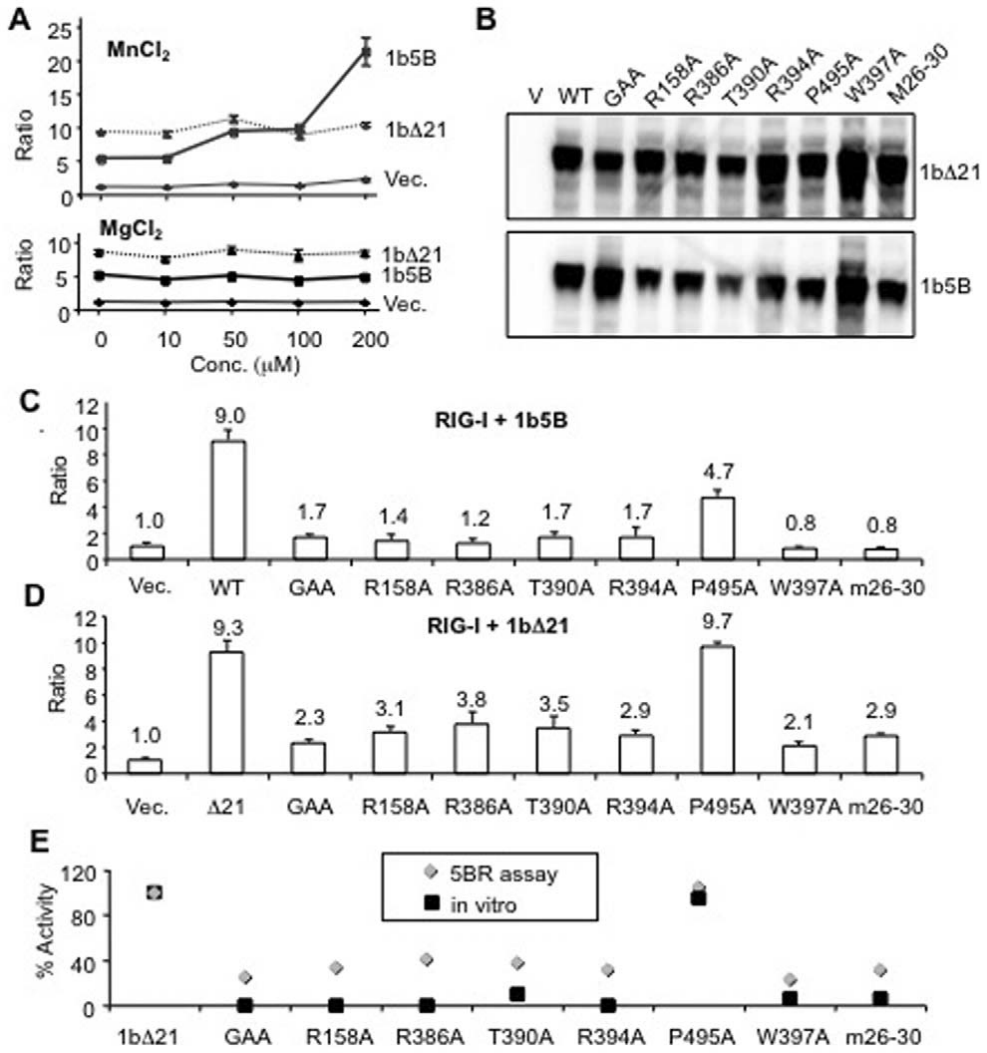
NS5B activity and the 5BR assay. A) Effects of Mn^2+^ (top panel) or Mg^2+^ (bottom panel) on the readout from the 5BR assay. The results from cells expressing 1b5B, 1bΔ21, and the empty vector (Vec.) are plotted with the means and standard deviation of three assays. The results were reproducible in six independent samples. B) Western blots showing steady state levels of the proteins from the HCV mutants. C and D) A comparison of the firefly and *Renilla* luciferase ratios in HEK 293T cells transfected to express the NS5B construct indicated at the bottom of the bar graphs along with RIG-I plasmid. The means and standard deviations from at least three independently tested samples are shown above the bars. E) Correlation of RNA synthesis in vitro by recombinant 1bΔ21 protein and the results from the 5BR assay expressing 1bΔ21. Data for RNA synthesis in the biochemical assays were taken from the results from Ranjith-Kumar et al. [26] and Chinnaswamy et al. [21] and normalized to that of the WT sample in the same experiment. In vitro RNA synthesis results were from variants of recombinant 1bΔ21 analyzed in the presence of only Mg^2+^. The data from the 5BR assay are derived from panel D above.

A feature of RNA synthesis by the 1b HCV polymerase is that Mn^2+^ can potently enhance de novo initiation [33]. To examine whether Mn^2+^ will affect the 5BR assay, we supplemented the medium with up to 200 µM MnCl_2_ (Fig. 2A, top graph). Higher Mn^2+^ concentrations decreased the *Renilla* luciferase levels even in the absence of NS5B expression, suggesting that there is toxicity. From 10 to 200 µM of Mn^2+^, however, we observed an up to a four-fold increase in the 5BR assay output in the presence of 1b5B. In vitro, the recombinant 1bΔ21 was stimulated by Mn^2+^ in the low millimolar range [33]. However, in the 5BR assay, the signal from 1bΔ21 did not increase until the Mn^2+^ concentration was in excess of 200 µM (Fig. 2A and data not shown). MgCl_2_ added to the medium did not result in an increase over the same concentrations (Fig. 2A, bottom graph). Finally, neither Mg^2+^ nor Mn^2+^ affected RIG-I signaling in the presence of exogenously provided 3PdsR24, suggesting that the observed increase in 1b5B activity in the presence of Mn^2+^ was due to increased polymerase activity (data not shown).

Residues in the recombinant 1bΔ21 protein have been shown to affect RNA synthesis in vitro, including the ability to initiate de novo or extend from a primer [26], [33], [34]. The 5BR assay offers an opportunity to compare the effects of these and other mutations in an intracellular environment. The mutants tested include those that affected NTP binding and catalysis (GAA, R158A, R386A, T390A, R394A), the low-affinity GTP binding site (P495A), the Δ1 loop that extends from the fingers to the thumb subdomain which regulates de novo initiation (m26–30) or the thumb subdomain that interacts with the Δ1 loop (W397A). Mutations m26–30 and W397A changed the conformations of the HCV polymerase and the ability to initiate by a de novo mechanism [21], [26], [35]. All mutant proteins were expressed in HEK 293T cells in the context of both full-length NS5B and the C-terminally truncated polymerase (Fig. 2B). In the 5BR assay, the effects of the mutations were highly similar with and without the C-terminal transmembrane helix (Fig. 2C & D). All mutants except P495A were significantly debilitated for the ability to activate RIG-I. We consistently observed no negative effect on reporter levels when P459A was tested in the Δ21 form, but found that reporter levels were at half the level of wild type when tested in the context of full-length NS5B (Fig. 2C & D). These results suggest that the membrane interaction can influence the effects of some mutations in NS5B. Additional examination of 1b5B interaction with the membrane will be presented later in this work.

Our laboratory had previously tested a large number of mutations in the context of recombinant 1bΔ21 protein [21], [26], [34]. Results from these mutants were found to be in excellent agreement with those from the 5BR assay (Fig. 2E). Furthermore, mutations that allowed the recombinant proteins to retain the ability to extend from a primed template but are defective for de novo initiated RNA synthesis (e.g. W397A, m26-30) were unable to increase luciferase production in the 5BR assay. These results suggest that RNA production and/or activation of RIG-I signaling require de novo initiation of RNA synthesis by NS5B in cells.

### Analysis with NS5B inhibitors

**Figure 3:**
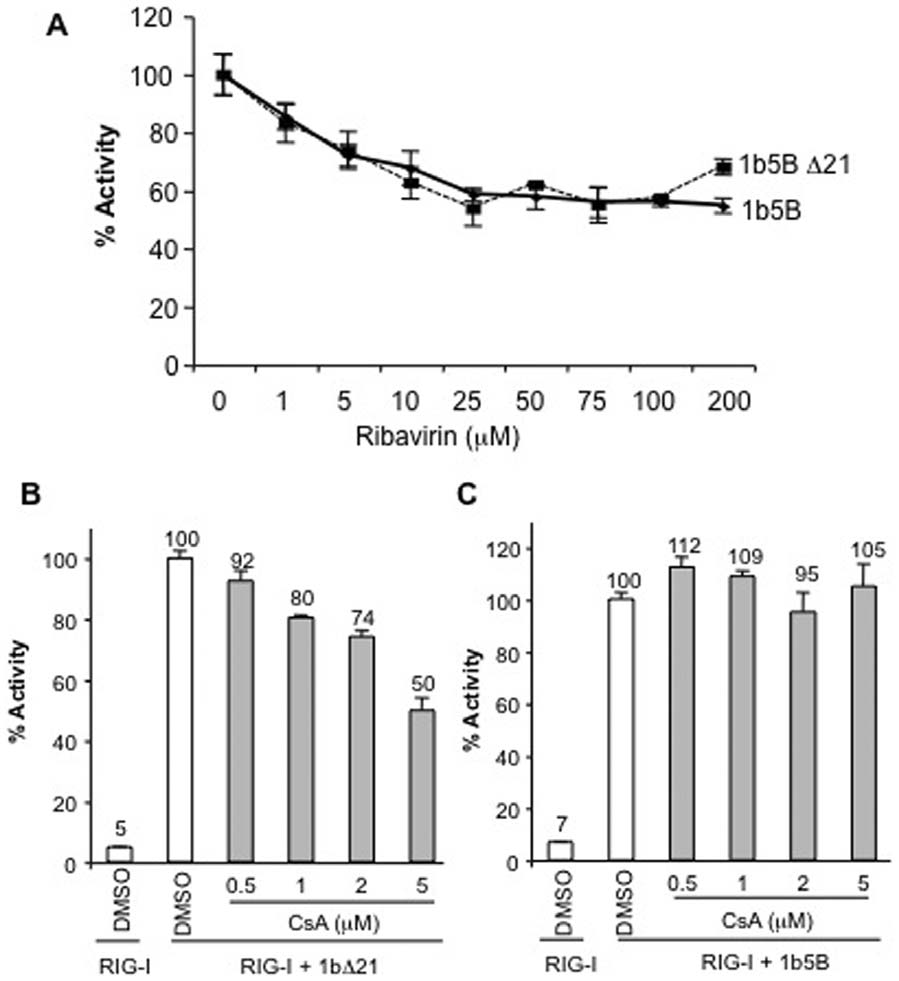
Effect of Ribavirin and cyclosporine A on the 5BR assay. A) Effects of Ribavirin on the 5BR results with the 1bΔ21 and 1b5B polymerases. Ribavirin was dissolved in DMSO at various concentrations and added to the cells 2 h after transfection of the expression plasmids to achieve the concentrations shown as well as a final DMSO concentration of 1%. The data were normalized to the ratio of the luciferase production of the sample treated with 1% DMSO. B) Effect of cyclosporine A on the 5BR assay expressing 1bΔ21. Cyclosporine A (CsA) was dissolved in DMSO in stocks and used at a final DMSO condition of 1% and the concentration of CsA shown on the horizontal axis of the graph. The samples treated with CsA are in grey. C) Effects of CsA on the 5BR assay expressing 1b5B.

The 5BR assay offers an opportunity to test inhibitors of NS5B in a cell-based assay [5]. Ribavirin, a purine analog, is an FDA approved anti-HCV drug that can modulate the immune response, inhibit host enzyme(s) and/or increase error rate of NS5B when incorporated into nascent RNA during replication [36], [37], [38]. In the 5BR assay, ribavirin up to 50 µM decreased the readout from the 5BR assay in a concentration-dependent manner (Fig. 3A). However, higher concentrations did not reduce the activity further and actually had less of an effect, suggesting that ribavirin solubility was decreased at higher concentrations. To confirm that the effect of ribavirin is through NS5B, RIG-I signaling with the ligand 3PdsR24 was analyzed. Ribavirin did not exhibit inhibitory effect on RIG-I signaling at 50 µM suggesting that Ribavirin inhibits NS5B activity and does not influence RIG-I signaling (Figure S1A). At 200 µM or higher concentrations of ribavirin, we observed a decrease in the *Renilla* luciferase values in the cells, suggesting that ribavirin had toxicity. This effect was observed in assays with both 1bΔ21 and 1b5B (Fig. 3A). Nonetheless, these results suggest that ribavirin can affect HCV polymerase activity.

Cyclosporine A and its derivatives have been proposed to inhibit HCV replication by interfering with the interaction of cyclophilins with NS5B, NS5A and/or NS3 [39], [40], [41]. The 5BR assay could thus be useful in determining whether NS5B activity requires cyclophilins in the absence of the other HCV proteins. Cyclosporine A showed a concentration-dependent inhibition of 1bΔ21 but surprisingly did not show any significant effect on 1b5B (Fig. 3B & C). Furthermore, cyclosporine A (10 µM) did not have affect 3PdsR24 dependent RIG-I signaling (Figure S1B). Taken together with the effect of MnCl_2_, these results suggest that the transmembrane domain of NS5B can influence both polymerase activity and sensitivity to cyclosporine.

**Figure 4:**
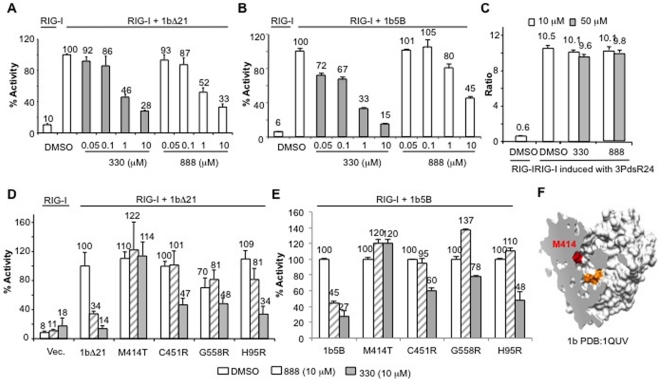
Inhibition of the 1b HCV polymerase by benzothiadiazine derivatives. A) BTDs 330 and 888 can inhibit the 1bΔ21 polymerase in a concentration-dependent manner. The BTD compounds are dissolved in DMSO and added to the media of the HEK 293T cells to the final concentrations shown on the horizontal axis. DMSO was at present at 1% final concentration. B) Effect of BTDs 330 and 888 on full length NS5B, 1b5B. The conditions for the assay are identical as described for 1bΔ21 polymerase. C) RIG-I was not inhibited by the BTDs. A RIG-I assay was performed in the absence of HCV polymerase using 3PdsR24 as the agonist. Where present, the compounds were tested at either 10 or 50 µM final concentration. D) Resistant mutations in 1bΔ21 can partially overcome the effects of BTD derivatives. “Vec.” denotes the cells transfected with the empty pUNO vector. The mutants tested in the 5BR assay were all constructed in the 1bΔ21 background and named only by the amino acids substituted. Results from cells treated with 10 µM of 888 or 330 are shown as grey striped and grey bars, respectively. All bars contain data from at least three independent samples. E) Sample tested with full length NS5B, 1b5B. The format of the results are identical to those from panel D. F) Location of residue 414 relative to the catalytic motif of the 1b Con 1 HCV polymerase. The 1b polymerase is shown in a cross section (PDB: 1QUV) and M414 is denoted in red color and motif C GDD from residues 317–319 is in gold.

Next, we tested benzothiadiazine (BTD) derivatives, non-nucleoside analogs that act at a cavity within the template channel of NS5B. Compound 888 was shown to be a potent inhibitor of HCV and its derivative, 330, had improved potency [20]. Both showed a concentration-dependent inhibition of RIG-I activation in the 5BR assay with 1bΔ21 with IC_50_s of ∼1 µM (Fig. 4A). 1b5B showed similar sensitivity to 330, but the IC_50_ for 888 was ∼10 µM (Fig. 4B). The compounds did not affect RIG-I signaling by the agonist 3PdsR24 even up to 50 µM, demonstrating that the inhibitory effect of the BTDs was on NS5B and not on the RIG-I signaling pathway (Fig. 4C).

Several mutations in NS5B rendered the 1b HCV replicons resistant to BTD derivatives, including M414T, C451R, G558R and H95R [42], [43]. These mutations were introduced into 1bΔ21 and tested in the 5BR assay along with compounds 330 and 888 (Fig. 4D). While the wild-type 1bΔ21 was inhibited by more than 70% by 10 µM of either 330 or 888, M414T was resistant to these compounds at either 10 or 20 µM (Fig. 4D and data not shown). Similar effect was observed with drug resistant mutants in 1b5B (Fig. 4E). The location of M414 relative to the active site is shown in Fig. 4F. Interestingly, C451R, G558R and H95R were partially resistant to 330 when compared to the wild-type 1bΔ21 and 1b5B, but were inhibited less well by 888. Taken together, these data indicate that the 5BR assay could be used to examine at least some HCV-specific inhibitors and suggest that BTDs 888 and 330 have overlapping, but not identical binding sites.

### Benzothiadiazines do not inhibit 2a NS5B

**Figure 5:**
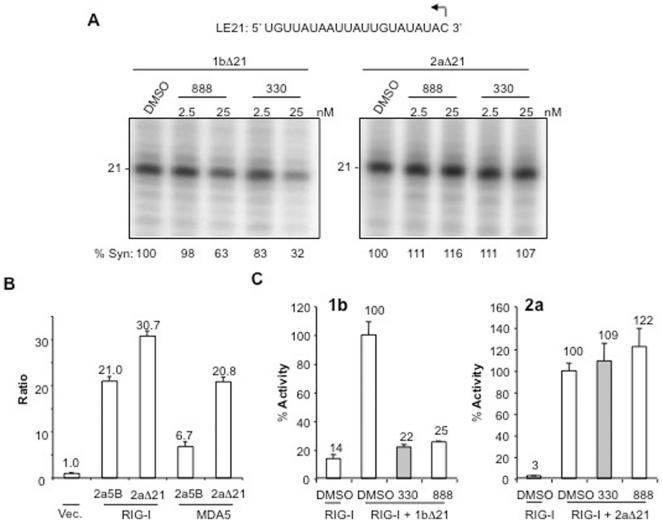
Differential effects of BTDs on the 1b and 2a HCV polymerases. A) RNA synthesis in vitro by the 1bΔ21 and the 2aΔ21 in the presence of two BTD derivatives. The sequence of the template (LE21) used is given on the top. The amount of the 21-nt de novo initiated product made was quantified and given below as percent synthesis relative to the sample treated with only DMSO. B) The full-length 2a NS5B (2a5B) and the 2aΔ21 polymerases can induce both RIG-I and MDA5 dependent luciferase production in the 5BR assay. The ratio shown in the graph denotes the ratio of firefly to *Renilla* luciferase activities in HEK 293T cells. C) Comparison of the effects of BTD derivatives 330 and 888 on the 1bΔ21 and the 2aΔ21.

Several of the HCV compounds are effective only to specific HCV genotypes [44]. The 2a polymerase has a glutamine at residue 414 instead of the methionine in 1b5B. If M414 is important for BTD binding in the 1b HCV polymerase, then the 2a polymerase should be less sensitive to BTDs. We first compared RNA synthesis in vitro by recombinant 2aΔ21 and 1bΔ21 in the absence or presence of the inhibitors. The reactions used a 21-nt RNA, LE21, as a template (Fig. 5A; [34]). The 2a polymerase is more active for de novo initiation than the 1b polymerase, consistent with previous results [43], [45]. However, while nanomolar concentrations of 888 or 330 significantly reduced RNA synthesis by the 1bΔ21, RNA synthesis by the 2aΔ21 was increased slightly in the presence of the BTD derivatives (Fig. 5A). These results encouraged us to test the genotype specificity of the BTDs in the 5BR assay. Constructs expressing 2a5B or 2aΔ21 activated luciferase production in the presence of either RIG-I or MDA5 two- to three-fold when compared to its 1b counterpart (Fig. 5B). Furthermore, like the 1b polymerases, signaling was more robust with RIG-I than MDA5.

Next, we determined whether the BTDs affect 2aΔ21 in the 5BR assay (Fig. 5C). Compounds 330 and 888 at 10 µM both caused a several-fold reduction of the readout from assays with 1bΔ21. In contrast, they increased the luciferase ratios slightly in cells expressing 2aΔ21 (Fig. 5C), consistent with the effects on RNA synthesis in vitro (Fig. 5A). These results demonstrate that BTDs do not inhibit the polymerase of the 2a genotype of HCV.

### The NS5B C-terminal transmembrane helix and the 5BR assay

**Figure 6:**
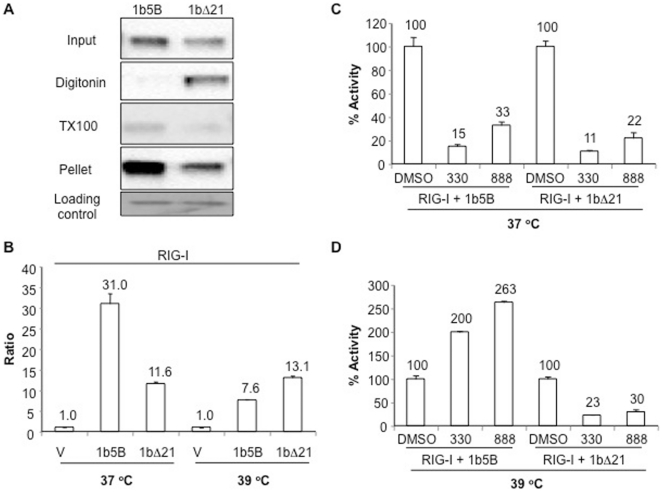
Effects of the NS5B transmembrane helix and sensitivity to BTDs. A) Subcellular fractionation to examine the localization of 1b5B and 1bΔ21. The cellular fractionation protocol was from that of Ramsby et al. [27]. The proteins were detected by Western blots probed with a monoclonal antibody to 1b NS5B. B) Effects of the NS5B transmembrane helix on the activity of HCV polymerase at 37°C and 39°C. Conditions for the assay were exactly the same except for the temperatures used. C and D) Effects of compound 330 and 888 (10 µM) on 1b5B and 1bΔ21 activity in the 5BR assay at 37°C (C) and 39°C (D).

While both the 1b and 2a HCV polymerases lacking the C-terminal transmembrane helices (TMHs) retain the ability to induce RIG-I signaling in HEK 293T cells, some differences in the results (e.g. response to Mn^2+^, effects of P495A and cyclosporine A) prompted us to examine whether the TMH will affect the outcome of the 5BR assay. To confirm that the transmembrane helix can affect NS5B localization, we performed a crude fractionation assay where the cells expressing either 1bΔ21 or 1b NS5B were first treated with digitonin to solubilize the plasma membrane to release the cytoplasmic proteins. The pellet was further treated with a buffer containing Triton X-100 to solubilize most of the intracellular membranes leaving a pellet fraction that contains insoluble materials. Each fraction was collected and subjected to Western blot analysis to identify 1b5B or 1bΔ21 (Fig. 6A). 1bΔ21 was more abundant in the digitonin-solubilized materials than 1b5B, indicating that the two proteins can at least partially localize to different subcellular locations.

To examine the effects of the TMH further, we compared the effects of 1b5B and 1bΔ21 in 293T cells grown at 37°C and 39°C (Fig. 6B). We have earlier observed that recombinant 1bΔ21 showed a temperature-dependent variation in activity [46]. Interestingly, in 5BR assay 1b5B showed a significant decrease in activity at 39°C while 1bΔ21 did not (Fig. 6B). To analyze this further, we tested BTD 330 and 888 on HEK 293T cells expressing 1b5B or 1bΔ21 at 37°C and 39°C (Fig. 6C and 6D). At 37°C, both 1bΔ21 and 1b5B were inhibited by 10 µM of 330 and 888 by more than 70%. At 39°C, 1bΔ21 was inhibited by BTDs while those with 1b5B not only were not inhibited, but resulted in slightly increased luciferase levels (Fig. 6D). Similar results were obtained when MDA5 was used instead of RIG-I in the assay (data not shown). These results suggest that either the TMH or its effect on polymerase membrane association by NS5B will influence its activity.

### Interaction with other HCV nonstructural proteins

The 5BR assay should allow us to analyze the interaction between NS5B and other HCV non-structural proteins. To explore this, we first expressed 1b5B along with the NS3 protein from the 1b genotype. The NS3 protein expressed was fused to residues 1 to 17 of NS4A (1b3-4AP, Figure S2A). However, due to the well-characterized effect of the wild-type NS3-4A protein abolishing RIG-I signaling by cleavage of adaptor IPS-1 [16], we also tested NS3-4A with a mutation in the NS3 protease active site in construct S135A. While NS3-4A inhibited RIG-I-dependent signaling, mutant S135A had no effect on 3PdsR24-dependent RIG-I signaling. S135A also had no effect on the 5BR assay expressing either 1b5B or 1bΔ21 (Figure S2A). Similar results were observed with NS3 fused to the intact NS4A protein (1b3-4AFL) or intact protein with the S135A substitution in the protease active site (Figure S2B). No significant changes were observed in the 5BR assay at higher concentrations of the S135A mutants (data not shown). Lastly, no obvious effects were observed with a construct expressing NS4B in the 5BR assay format (Figure S2C). These data suggest that HCV NS3, 4A and 4B do not affect RNA synthesis by NS5B in the 5BR assay.

**Figure 7:**
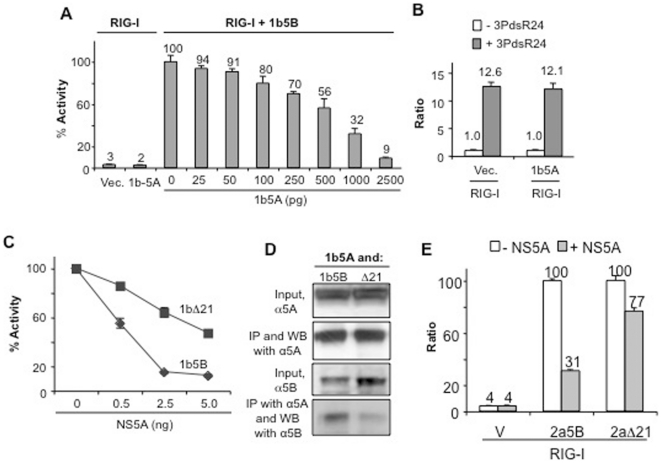
Effects of NS5A on the 5BR assay. A) Inhibition of the 5BR assay by increasing amount of plasmids expressing NS5A. B) The NS5A protein does not affect RIG-I signaling in the absence of NS5B. “Vec.” denotes samples transfected with the empty vector and “1b5A” corresponds to cells transfected to express NS5A. All samples expressed the RIG-I protein. C) A comparison of the sensitivity of 1b5B and 1bΔ21 to inhibition by NS5A. Each point was analyzed in triplicate and the standard deviation for several of the points are small. D) Co-immunoprecipitation of NS5B with NS5A and the effects of the membrane-spanning domains. Detergent-solubilized lysates from HEK 293T cells expressing the proteins labeled above the gel image were first subjected to immunoprecipitation (IP) with mAb against 1b5A, then subjected to Western blots (WB) with antibodies listed to the left of the gel images. Input controls for 1b5A and 1b5B are also shown. E) The 1b NS5A also inhibited the activity of the full-length (2a5B) or C-terminally truncated (2aΔ21) 2a NS5B. As with the 1b polymerases, inhibition was greater with the full-length 2a5B.

Interestingly, co-expression of NS5A (henceforth referred to as 1b5A) inhibited the output of the 5BR assay in a concentration-dependent manner (Fig. 7A). When the RIG-I agonist 3PdsR24 was transfected into the cells, robust luciferase activity was observed in cells expressing 1b5A, indicating that 1b5A inhibited RNA synthesis by NS5B, not the RIG-I signaling (Fig. 7B). NS5A also did not affect RIG-I signaling with heterogeneous poly(I:C) and a 60-nt long triphosphorylated ssRNA, shR9 (data not shown). The addition of 1b5A to biochemical RNA synthesis reactions containing variants of NS5B was reported to stimulate RNA synthesis at substoichiometric levels and to dramatically inhibit RNA synthesis at higher levels [47], [48]. Transfection of substoichiometric levels of the NS5A construct did not result in an increase in the output from the 5BR assay in our hands (Fig. 7A).

Both NS5B and NS5A are associated with membranes. To examine whether membrane association will affect their interaction, we compared NS5A's effect on 1bΔ21 versus 1b5B using the 5BR assay. At all concentrations tested, 1bΔ21 was inhibited less well than that with 1b5B (Fig. 7C). These results suggest that that TMH and/or membrane association by NS5B contribute to interaction with NS5A. To test this notion, we attempted to co-immunoprecipitate 1b5A with either 1b5B or 1bΔ21. The results from three independent experiments consistently showed that at least two-fold more 1b5B co-precipitated with 1b5A than did 1bΔ21 (Fig. 7D). We also assessed the effect of 1b5A on 2a5B versus 2aΔ21 to determine whether this interaction is dependent on the HCV genotype (Fig. 7E). Similar to its 1b counterpart, the 1b5A inhibition of 2a5B was inhibited more efficiently than that of 2aΔ21. These results suggest that efficient NS5A/5B interaction may require TMH of NS5B, but so far there is no evidence of genotype–specific interaction between the NS5A and NS5B. Furthermore, since NS5A was not changed in these assays, these results suggest that RNA binding by NS5A is not primarily responsible for the inhibition of the NS5B activity.

### The 5BR assay in Huh7 cells

**Figure 8:**
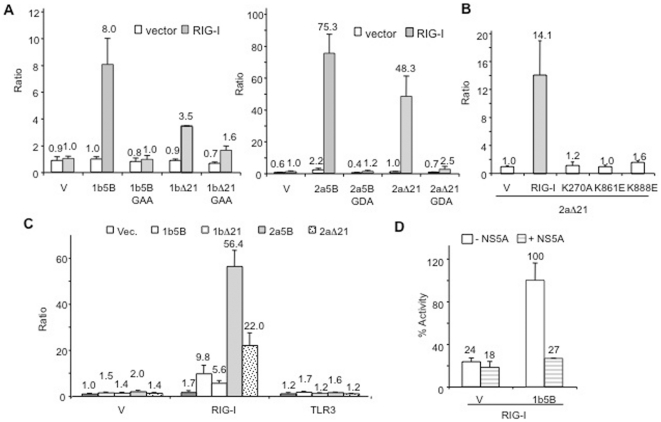
The 5BR assay in Huh7 cells. A) Ratio of firefly to the *Renilla* luciferase production in transiently-transfected Huh7 cells. All of the cells were transfected to express RIG-I or vector control, an IFN-β luciferase, the *Renilla* luciferase driven by CMV promoter, and the 1b or 2a polymerases, as shown. The mean and standard deviation of the results are shown above the bars. Constructs identified with GAA or GDA contain mutation in the catalytic pocket of the 1b or 2a polymerases, respectively. B) Mutations in the co-expressed RIG-I that affected signaling prevented activation of the IFN-β luciferase reporter in Huh7 cells. C) HCV polymerases co-expressed with RIG-I and MDA5, but not TLR3, resulted in activation of IFN-β luciferase reporter in Huh7 cells. D) 1b NS5A can inhibit NS5B-dependent RIG-I signaling in Huh7 cells.

We transfected the plasmids for the 1b and 2a NS5Bs into Huh7 cells to determine whether the assay can work in liver hepatocytes. Both the 1b and the 2a NS5B proteins were found to activate signaling by the co-expressed RIG-I protein (Fig. 8A). Consistent with the results in HEK 293T cells, the 2a5B-expressing cells produced higher activity than the 1b5B (Fig. 8A). Furthermore, active-site mutants (GAA for 1b and GDA for 2a) were incapable of activating reporter activity in Huh7 cells and cells expressing vector alone instead of RIG-I or RIG-I mutants did not show an increase in luciferase reporter activity (Fig. 8A & B). Also consistent with the results from HEK 293T cells, MDA5 could replace RIG-I for activation of signaling, but TLR3 could not (Fig. 8C). We note that in contrast to HEK 293T cells, full-length NS5B was more active than the C-terminal truncated version in Huh7 cells. Finally, we observed that co-expression of the 1b5A inhibited 1b5B in Huh7 cells in a manner similar to results from HEK 293T cells (Fig. 8D). These results show that the 5BR assay can work in either Huh7 or HEK 293T cells.

### The 5BR assay with NS5Bs from all six major HCV genotypes

**Figure 9:**
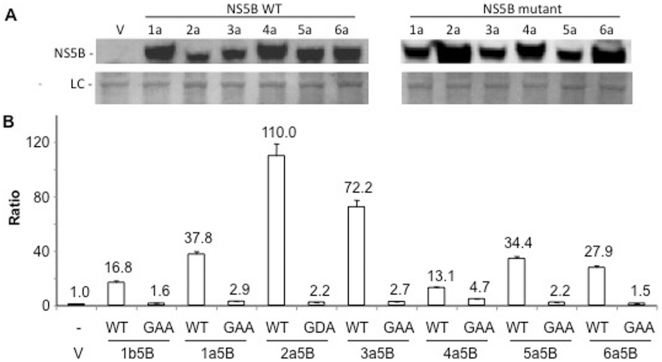
5BR assay with NS5Bs from the six major HCV genotypes. A) A Western blot demonstrating the expression of the NS5B proteins from all six HCV genotypes analyzed in the 5BR assay. The proteins were expressed in HEK 293T cells with C-terminal histidine tags and then probed with a monoclonal antibody detecting the tag. B) Results from the 5BR assay for the HCV polymerases. Corresponding catalytic mutants (denoted by GAA) were also tested as controls. The means and standard deviations of each result are shown. The results are representative of three independent assays.

We have observed that NS5Bs from HCV 1b and 2a genotypes could induce RIG-I signaling (Fig. 1 & 5). To test whether NS5Bs from the other genotypes could induce RIG-I, we cloned NS5B from genotypes 1a, 3, 4 and 6a and their corresponding catalytic mutants in pUNO vector. All proteins were expressed with a hexa-his tag at the C-terminus and their expression in HEK293T cells was confirmed by Western blot (Fig. 9A). In the 5BR assay, all six NS5Bs activated RIG-I signaling while their counterparts with active site mutation did not (Fig. 9B). The 2a5B and the 3a5B had robust activity while the 1a, 5a and 6a NS5Bs showed similar activity. Lastly, the 4a NS5B and the 1b5B had more modest activities. This suggests that 5BR assay could be used to study and compare the activities of the NS5B from all six genotypes of HCV that lack an efficient cell-based assay system.

## Discussion

There remains a significant need to develop effective inhibitors targeting the HCV polymerase as well as to understand the many roles of the HCV polymerase in the infection process. We have developed a cell-based assay for HCV NS5B from all genotypes named the 5BR assay that couples RNA synthesis to the detection and subsequent reporter production by innate immune receptor RIG-I. This assay format does not activate signaling by Toll-like receptor 3, but can activate the RIG-I-like receptor, MDA5. MDA5 has been reported to require RNAs that are kilobasepairs in length [24], [32], this suggests that at least some of the HCV polymerase products could be quite long. Mutations of the residues in NS5B that are essential for RNA polymerization reduced readout from the assay. The 5BR assay was validated for compounds previously characterized to specifically target the 1b HCV replicons. Mutations that conferred resistance to benzothiadiazines in HCV replicons are resistant in the 5BR assay format. The 5BR assay could also duplicate an inhibitory activity of NS5A when it is co-expressed with NS5B. Intriguingly, the transmembrane helix in NS5B could be deleted without affecting the ability to activate RIG-I signaling, but the lack of the transmembrane helix decreased the interaction with NS5A, how divalent metals affect RNA synthesis, and sensitivity to cyclosporine A.

This assay provides an advantage to existing subgenomic HCV replicon assay and biochemical assays for HCV polymerase activity. It could be used to validate whether compounds identified to be effective in the replicon assays, targets the HCV polymerase. This assay could also be performed with multiple cells types as opposed to hepatocytes for subgenomic replicon and JFH1 infection assays. When the polymerases are expressed from integrated transgenes, this assay should be suitable for high-throughput screening efforts to identify HCV polymerase inhibitors. The assay is also advantageous when compared to biochemical polymerase assays since polymerase activity is examined under physiological conditions. In addition, the assay does not require extensive purification of the polymerase prior to assessing the properties of mutations in the polymerase. The availability of polymerases from all six genotypes of HCV (Fig. 9) can allow examination of genotype-specific effects of cellular factors and polymerase inhibitors.

Moriyama et al [49] have shown that expression of HCV NS5B in HepG2 cells could induce IFN-β in a TLR3-dependent manner and NS4A, NS4B and NS5A inhibited this activation. In our 5BR assay, TLR3 is not activated by 1b and 2a NS5Bs in either HEK 293T or Huh7 cells (Fig. 1D and Fig. 8C). TLR3 recognizes the internal structure within a dsRNA, and recognition strongly requires acidic conditions, like those found in endosomes [25], [50], [51]. We believe that RNAs synthesized by NS5B are inaccessible to TLR3 harbored in the endosomes and that there is a strong dependence on RIG-I as well as de novo initiation by the HCV polymerases to produce the ligand to activate innate immune signaling. Of the HCV nonstructural proteins we tested (NS3, NS3-4A, NS4B, and NS5A), only NS5A affected NS5B activity (Figure S2 & Fig. 7). NS5A is a logical candidate to reconstitute an effect on NS5B since it can act in trans of HCV replicase [52], [53]. We did observe an effect of protease-active NS3 and NS3-4A on the assay, but this is likely due to NS3 protease cleaving the RIG-I adaptor IPS-1 (which we confirmed to occur in Western blots) and a mutation in the NS3 protease activity prevented any obvious effect on the 5BR assay (Figure S2). NS4B is the other HCV nonstructural protein that could act in trans of the HCV replicase [49]. However, we did not observe modulation of NS5B-dependent RIG-I signaling by NS4B. Since this is a negative result, it is best to not over-interpret its relevance. In summary, there are obvious differences and utilities between our assay and the one of Moriyama et al. [49].

There are several lines of evidence suggesting that a de novo initiated RNA produced by the HCV polymerase is responsible for activating RIG-I. First, all mutants that were defective for de novo initiation of RNA synthesis in the absence of Mn^2+^ in biochemical assays were unable to efficiently induce RIG-I signaling (Fig. 2C–E). Second, mutants that are capable of extension from a primer were incapable of inducing reporter activity in the 5BR assay. Third, the addition of Mn^2+^, a cofactor known to increase de novo initiation in vitro, increased the NS5B-dependent luciferase production (Fig. 2A). Fourth, the HCV 2aΔ21, which is more active for de novo initiated RNA synthesis than 1bΔ21 in vitro, is better at activating luciferase production in the 5BR assay (e.g., compare Fig. 1B and 5B, and Fig. 8A). Fifth, an inhibitor of de novo initiation in vitro, BTD [20] inhibited output from the 5BR assay (Fig. 4A). These results should allow future analysis of HCV polymerase activities in cells. The excellent correlation between RNA synthesis in biochemical assays and the 5BR assay (Fig. 2E) also validates the use of biochemical approaches to studying properties of HCV polymerase for RNA synthesis in vitro.

Despite repeated effort, we have yet to observe a specific RNA product made by NS5B in this assay. The template sequence coding for NS5B contains a cis-acting replication element (CRE) that allows the cis-preferential use of the HCV RNA for replication [54]. Mutations in key nucleotides in the CRE that abolished HCV replicon replication but did not alter the NS5B protein sequence had no effects on the 5BR assay output in HEK 293T and Huh 7 cells (Figure S3). Extraction of the total RNAs from cells active for the 5BR assay did not reveal obvious differences from those of control cells. Furthermore, attempts to extract RNAs associated with RIG-I after immunoprecipitation of RIG-I generated smears of RNA suggesting that there are multiple RNAs being recognized by RIG-I (data not shown). We currently believe that the RNAs produced by the HCV polymerases are heterogeneous and may use templates from cellular RNAs, as is the case in vitro. The identification of the template(s) for the 5BR assay would require more extensive analysis and could be the subject of an independent study. However, regardless of whether one or many templates are used by the HCV polymerases, the assay will be valuable for a number of applications, as described below.

### NS5B inhibitors

The 5BR assay and also the assay where MDA5 substitutes for RIG-I can be used to determine whether NS5B is the target. The availability of the polymerases from all six major HCV genotypes in the assay could also allow rapid determination of the genotype-specific effects of drug candidates. These assays could also be used to examine the cellular factors that could modulate the HCV polymerase activity. For example, our observation that cyclosporine A can inhibit luciferase levels in the 5BR assay is in support of the report that the HCV polymerase requires cyclophilins for function [40], [55].

The benzothiadiazines 888 and 330 have nanomolar IC_50_s for RNA synthesis by the 1b NS5B in vitro and in the subgenomic replicon systems [20], [56]. The compounds have low micromolar IC_50_s in the 5BR assay (Fig. 4A). The difference between the inhibitory effects is likely due to issues with the compounds gaining access to the HCV polymerase in HEK 293T cells. Residue M414 in the HCV polymerase template channel that conferred resistance to 888 was phenocopied by the 5BR assay (Fig. 4C). We have also showed that the 2a polymerase, with a glutamine at residue 414, is not sensitive to BTDs in vitro or in the 5BR assay format (Fig. 5). In fact, the 2a polymerase may even be stimulated to higher levels of RNA synthesis by 888. The 5BR assay thus can allow examination of NS5B inhibitors in the absence of other HCV drug targets in a cellular environment. We note that this provides an advantage over the biochemical assays since the uptake of inhibitors by cells is a major issue in the efficacy of inhibitors.

Ribavirin only had a modest inhibitory effect on RNA synthesis in the 5BR assay even when added to cells at 50 µM (Fig. 3A & B). Higher ribavirin concentrations may be needed to compete with the cellular NTP pool for incorporation into the polymerase products. It is also possible that HEK 293T cells may not efficiently convert ribavirin to the triphosphate form to concentrations needed to affect RNA synthesis by NS5B (Fig. 3A & B). If ribavirin or nucleoside analogs caused chain termination, we note that RIG-I could be activated by RNAs that ca. 20-nt [24], [32].

### Transmembrane helix, polymerase conformation, and the 5BR assay

We observed a number of differences in the activity of NS5B with and without the C-terminal transmembrane helix. First, MnCl_2_-dependent activation of signaling in the 5BR assay was observed only with 1b5B and not with 1bΔ21 (Fig. 2A). Second, the P495A mutation had a differential effect in activating RIG-I based on the protein (Fig. 2C & D). Third, the presence of the NS5B TMH affected the inhibitory activity of cyclosporine A (Fig. 3B & C). Fourth, BTDs showed differential effect on 1b5B at 37 and 39°C (Fig. 6C & D). The effect of the TMH could be due to association of the polymerase with the cellular membranes and/or an effect on the active site of the HCV polymerase. The sequence adjacent to the TMH lines the template channel of the HCV polymerase [35], [57], [58]. Oh et al. [59] also demonstrated that the full-length 1b NS5B could bind RNA better than 1bΔ21, consistent with an effect on the template channel. We surmise that the effect on the template channel may have contributed to altered responses to Mn^2+^ and could have influenced the effect of cyclophilins that contributes to template binding by the HCV polymerase [55], [60]. The 5BR assay format could be used to further dissect the requirements for how the C-terminal TMH can influence polymerase activity within the cell.

### Interaction with NS5A

1b5A inhibited both 1b5B and 2a5B activity in the 5BR assay in a concentration-dependent manner (Fig. 7A and 7E). With regard to the biological relevance of this interaction, it is known that NS5A and 5B form a complex [47], [61]. It is possible that the interaction of these two proteins in the absence of all of the components of the HCV replicase could lead to a repression of RNA synthesis. This has been observed in biochemical assays where higher concentrations of NS5A were present [47], although lower concentrations of NS5A could increase both initiation and elongation by the NS5B protein [48]. It is also likely that activators as well as repressors must regulate HCV RNA synthesis and NS5A could serve to repress HCV RNA synthesis, should excess RNA synthesis trigger innate immune responses.

In terms of mechanism, NS5A could act through binding to dsRNA or an interaction with NS5B. Our results suggest that a more direct interaction between NS5A and NS5B is important for the inhibition rather than RNA sequestration. First, if 1b5A acts through binding RNAs, it should have inhibited the activation of RIG-I by binding to the exogenously provided RIG-I agonists. Second, RNA binding by NS5A should be unaffected by the expression of 1b5B or the 1bΔ21. Instead, we observed that 1bΔ21 was not inhibited to the same level as 1b5B, suggesting that membrane association of 5B contributes to inhibition by 5A (Fig. 7C). In support of the direct interaction model, we showed that 1b5B co-immunoprecipitated with NS5A better than it did with 1bΔ21 (Fig. 7D). At present, we have not been able to detect a stimulation of NS5B RNA synthesis by low levels of NS5A in our assay [47], [48]. It is possible that the TMH of NS5B could influence NS5A-NS5B through protein-protein interactions directly or through the formation of a complex that involves cellular membranes. The 5BR assay should be useful to further dissect how the two proteins interact. Furthermore, the negative effects of NS5A on NS5B could be used as a basis to identify inhibitors that prevent their interaction.

### Future perspectives

The 5BR assay format could have a number of applications. First, it could be used to study the interaction of viral polymerases with viral or cellular factors that could modulate polymerase activity. Second, the assay provides a way to characterize RIG-I and MDA5 signaling without the use of exogenous agonists. Third, the 5BR assay could be used to screen for inhibitors of polymerases in the context of mammalian cells. Fourth, it can be used to examine how motifs in the HCV polymerase can affect function. This assay can be developed for use in a high throughput format once the polymerase has been integrated into the cells under an inducible promoter. One application that could be particularly useful is that the assay could be used for viruses that cannot replicate efficiently in cultured cells.

## Supporting Information

Figure S1 **Effects of inhibitors on signaling by RIG-I.** The concentrations of Ribavirin and Cyclosporin A (CsA) tested are those that were able to inhibit the 5BR assay. A) Effects of Ribavirin. B) Effects of CsA.
(DOC)

Figure S2 **Effects of co-expressing NS3-4A, NS3-4A mutant S135A, or NS4B on the 5BR assay.** A) Analysis of NS3 fused to 4A peptide and NS3 protease active site mutant S135A on the 5BR assay. The S135A mutation is in a catalytic residue of the NS3 protease domain. The constructs tested express full-length NS3, but only the peptide from NS4A can enhance NS3 protease activity. B) Analysis of a NS3 and full-length NS4A fusion protein as well as mutant S135A on the 5BR assay. C) Analysis of the NS4B protein on the 5BR assay. White and grey bars correspond to RIG-I signaling in the absence and presence of transfected RIG-I agonist 3PdsR24, respectively. The ratios of the plasmids encoding the proteins used are given below the bars.
(DOC)

Figure S3 **Analysis of** ***cis*** **-acting replication element (CRE) in the sequence encoding NS5B. A.** Predicted secondary structure of the RNA corresponding to NS5B 3′ end coding for AA 539–591. The sequence and predicted secondary structure of the CRE is shown on the right and derived from the analysis of You et al. [52]. Mutations U71C and C90A inhibited replication of HCV replicon but U86G did not have any significant effect [52]. B&C) Effects of the mutations in the CRE element on the 5BR assay performed with HEK 293T cells (B) and Huh7 cells (C).
(DOC)
