# Supplementary material for: A Cell-Based Assay for RNA Synthesis by the HCV Polymerase Reveals New Insights on Mechanism of Polymerase Inhibitors and Modulation by NS5A
Source: PLoS One. 2011 Jul 22;6(7):e22575. doi: 10.1371/journal.pone.0022575 (PMC3142182; doi:10.1371/journal.pone.0022575)
Supplement: Figure S1 — Effects of inhibitors on signaling by RIG-I. The concentrations of Ribavirin and Cyclosporin A (CsA) tested are those that were able to inhibit the 5BR assay. A) Effects of Ribavirin. B) Effects of CsA. (DOC) [file pone.0022575.s001.doc]

**sFig. 1**. Effects of inhibitors on signaling by RIG-I. The concentrations of Ribavirin and Cyclosporin A (CsA) tested are those that were able to inhibit the 5BR assay. A) Effects of Ribavirin. B) Effects of CsA.
