# Supplementary material for: A Cell-Based Assay for RNA Synthesis by the HCV Polymerase Reveals New Insights on Mechanism of Polymerase Inhibitors and Modulation by NS5A
Source: PLoS One. 2011 Jul 22;6(7):e22575. doi: 10.1371/journal.pone.0022575 (PMC3142182; doi:10.1371/journal.pone.0022575)
Supplement: Figure S2 — Effects of co-expressing NS3-4A, NS3-4A mutant S135A, or NS4B on the 5BR assay. A) Analysis of NS3 fused to 4A peptide and NS3 protease active site mutant S135A on the 5BR assay. The S135A mutation is in a catalytic residue of the NS3 protease domain. The constructs tested express full-length NS3, but only the peptide from NS4A can enhance NS3 protease activity. B) Analysis of a NS3 and full-length NS4A fusion protein as well as mutant S135A on the 5BR assay. C) Analysis of the NS4B protein on the 5BR assay. White and grey bars correspond to RIG-I signaling in the absence and presence of transfected RIG-I agonist 3PdsR24, respectively. The ratios of the plasmids encoding the proteins used are given below the bars. (DOC) [file pone.0022575.s002.doc]

**sFig. 2**. Effects of co-expressing NS3-4A, NS3-4A mutant S135A, or NS4B on the 5BR assay. A) Analysis of NS3 fused to 4A peptide and NS3 protease active site mutant S135A on the 5BR assay. The S135A mutation is in a catalytic residue of the NS3 protease domain. The constructs tested express full-length NS3, but only the peptide from NS4A can enhance NS3 protease activity. B) Analysis of a NS3 and full-length NS4A fusion protein as well as mutant S135A on the 5BR assay. C) Analysis of the NS4B protein on the 5BR assay. White and grey bars correspond to RIG-I signaling in the absence and presence of transfected RIG-I agonist 3PdsR24, respectively. The ratios of the plasmids encoding the proteins used are given below the bars.
