# Supplementary material for: A Cell-Based Assay for RNA Synthesis by the HCV Polymerase Reveals New Insights on Mechanism of Polymerase Inhibitors and Modulation by NS5A
Source: PLoS One. 2011 Jul 22;6(7):e22575. doi: 10.1371/journal.pone.0022575 (PMC3142182; doi:10.1371/journal.pone.0022575)
Supplement: Figure S3 — Analysis of cis -acting replication element (CRE) in the sequence encoding NS5B. A. Predicted secondary structure of the RNA corresponding to NS5B 3′ end coding for AA 539–591. The sequence and predicted secondary structure of the CRE is shown on the right and derived from the analysis of You et al. [52]. Mutations U71C and C90A inhibited replication of HCV replicon but U86G did not have any significant effect [52]. B&C) Effects of the mutations in the CRE element on the 5BR assay performed with HEK 293T cells (B) and Huh7 cells (C). (DOC) [file pone.0022575.s003.doc]

**sFig. 3**. Analysis of *cis*-acting replication element (CRE) in the sequence encoding NS5B. **A.** Predicted secondary structure of the RNA corresponding to NS5B 3’ end coding for AA 539-591. The sequence and predicted secondary structure of the CRE is shown on the right and derived from the analysis of You et al. [52]. Mutations U71C and C90A inhibited replication of HCV replicon but U86G did not have any significant effect [52]. B&C) Effects of the mutations in the CRE element on the 5BR assay performed with HEK 293T cells (B) and Huh7 cells (C).
